# Lower urinary tract symptoms and sexual dysfunction in men with multiple sclerosis

**DOI:** 10.6061/clinics/2019/e713

**Published:** 2019-03-05

**Authors:** André Luiz Farinhas Tomé, Eduardo P Miranda, José de Bessa Júnior, Carlos Alberto Bezerra, Antônio Carlos Lima Pompeo, Sidney Glina, Cristiano Mendes Gomes

**Affiliations:** I Divisao de Urologia, Faculdade de Medicina do ABC, Santo Andre, SP, BR; II Divisao de Urologia, Associacao Brazileira de Esclerose Multipla (ABEM), Sao Paulo, SP, BR; III Divisao de Urologia, Faculdade de Medicina FMUSP, Universidade de Sao Paulo, Sao Paulo, SP, BR

**Keywords:** Bladder Dysfunction, Demyelinating Diseases, Erectile Dysfunction, Lower Urinary Tract Symptoms, Multiple Sclerosis, Sexual Symptoms

## Abstract

**OBJECTIVES:**

To assess the prevalence and interrelationship between lower urinary tract symptoms and sexual dysfunction in men with multiple sclerosis (MS).

**METHODS:**

In a cross-sectional study, we evaluated 41 men (mean age 41.1±9.9 years) with MS from February 2011 to March 2013, who were invited to participate irrespective of the presence of lower urinary tract symptoms or sexual dysfunction. Neurological impairment was assessed with the Expanded Disability Status Scale; lower urinary tract symptoms were evaluated with the International Continence Society male short-form questionnaire, and sexual dysfunction was evaluated with the International Index of Erectile Function. All patients underwent transabdominal urinary tract sonography and urine culture.

**RESULTS:**

The mean disease duration was 10.5±7.3 years. Neurological evaluation showed a median Expanded Disability Status Scale score of 3 [2-6]. The median International Continence Society male short-form questionnaire score was 17 [10-25]. The median International Index of Erectile Function score was 29 [15-46]. Twenty-nine patients (74.4%) had sexual dysfunction as defined by an International Index of Erectile Function score <45. Voiding dysfunction and sexual dysfunction increased with the degree of neurological impairment (r=0.02 [0.02 to 0.36] *p*=0.03 and r=-0.41 [-0.65 to -0.11] *p*=0.008, respectively). Lower urinary tract symptoms and sexual dysfunction also displayed a significant correlation (r=-0.31 [-0.56 to -0.01] *p*=0.04).

**CONCLUSIONS:**

Most male patients with MS have lower urinary tract symptoms and sexual dysfunction. The severity of the neurological disease is a predictive factor for the occurrence of voiding and sexual dysfunctions.

## INTRODUCTION

Multiple sclerosis (MS) is the most common neurological disease in young adults, with peak incidence between ages 30 and 35 1. It is a chronic and progressive immune-mediated inflammatory disease that affects the central nervous system. MS may manifest different neurological symptoms and usually progresses to loss of physical and cognitive capabilities 2,3.

Lower urinary tract symptoms (LUTS) are frequent in MS patients 4. After 10 years of disease activity, it is estimated that more than 90% of MS patients report LUTS, and 70-80% have changes in voiding patterns during follow-up 4. Furthermore, LUTS are observed as the first manifestation of MS in 2 to 10% of patients 5. The impact of LUTS is significant, with impairment of quality of life (QOL), social functioning and sexual health 6,7. It is essential to understand the extent of the problem and to provide effective treatment for the affected individuals.

Sexual dysfunction (SD) is also highly prevalent in patients with MS and is often overlooked 8,9. SD may have a tremendous negative impact on the QOL, especially considering that many patients are young individuals 8-10. It is estimated that 50-90% of men with MS have SD, including erectile dysfunction (ED), ejaculatory disorders, orgasmic dysfunction and decreased libido 8,9.

An association seems to exist between LUTS and SD in patients with neurological impairment 8,11. This association is likely due to the common autonomic innervation of the genitourinary tract 12. Even though the prevalence and impact of LUTS and SD have been described in the MS population, the association between these distinct entities and the neurological status have not been properly investigated. Moreover, most studies have focused on female patients, who are more commonly affected by the disease. We performed a cross-sectional study to determine the prevalence and burden of LUTS and SD in men with MS. In addition, we investigated their interrelationships and associations with neurological disability.

## MATERIALS AND METHODS

### Study Population

In a cross-sectional study conducted from February 2011 to March 2013, we recruited consecutive male MS patients who presented at our Neurology Clinic. Participants were adults aged ≥21 years with a clinical diagnosis of MS according to the McDonald criteria 13. To be included in the present study, all subjects had to be in remission for at least 6 months since the last exacerbation episode. The exclusion criteria were prostate volume ≥30 g (as measured by transabdominal sonography), urinary retention requiring an indwelling catheter, previous history of pelvic or prostate surgery, previous pelvic radiotherapy, presence of bladder stones, urethral stricture, pharmacological treatment with 5-alpha reductase inhibitors or phosphodiesterase type 5 inhibitors (PDE5), severe cognitive or neuromuscular impairment and signs of disease exacerbation.

### Neurological Assessment

The severity of neurological impairment was assessed with the Expanded Disability Status Scale (EDSS, ranging from 0 to 10), which was performed by neurologists who specialized in demyelinating diseases. EDSS scores are based on measures of impairment in eight functional systems: visual, pyramidal, sensory, cerebellar, bowel and bladder, cerebral, brainstem and ambulation. EDSS steps 5.0 to 9.5 are characterized by impaired walking. An EDSS score >8 refers to a bedridden patient, which indicates severe cognitive and/or neuromuscular impairment; therefore, this value was used as a cutoff to participate in the study. The duration of MS was defined as the time since the first onset of neurological symptoms.

### LUTS Assessment

LUTS were evaluated with the abbreviated questionnaire of the International Continence Society for males (ICSmSF), based on the ICS definitions of LUTS 14. This is a standardized instrument that collects information on hesitancy, straining to void, slow stream, intermittency, incomplete emptying, terminal dribble, urgency, urge incontinence, stress incontinence, unpredictable urinary incontinence, nocturnal enuresis, frequency and nocturia. It provides not only a total ICSmSF score but also scores for specific LUTS categories: voiding (ICSV), incontinence/storage (ICSI), and LUTS-related QOL subscores 14. The answers are displayed as a five-point Likert scale including “never” (0), “occasionally” (1), “sometimes” (2), “most of the time” (3) and “all of the time” (4). Each LUTS was considered present whenever “sometimes”, “most of the time” or “always” was indicated 15. Increased urinary frequency was defined as intervals ≤2 hours 14. Nocturia was defined as two or more voids per night 16. QOL was scored from 0 to 3 according to the degree of burden associated with LUTS: 0 (not at all), 1 (mild), 2 (moderate) and 3 (severe).

The workup also included serum creatinine and prostate specific antigen (PSA) levels, urine culture and transabdominal sonography for evaluation of the kidneys, bladder, prostate and postvoid residual urine (PVR). Patients with a positive culture were treated prior to completing the ICSmSF questionnaire.

### Sexual Function Assessment

Sexual function was evaluated using the International Index of Erectile Function (IIEF-15) 17, which contains 15 questions evaluating five domains of sexual function: erectile function (EF), orgasmic function, sexual desire, satisfaction with sexual intercourse and overall satisfaction, with total scores ranging from 5 to 75. Dysfunctions in each domain were classified according to specific scoring as no dysfunction, mild, mild to moderate, moderate, and severe dysfunction 17. Severe impairment is associated with the lowest scores. Patients with total IIEF scores ≤45 were considered as having SD 17. EF was assessed by the IIEF-5 questionnaire, which is derived from the IIEF-15. Individuals with an IIEF score ≥17 were considered no to have significant ED 18,19.

### Associations among Neurological Impairment, LUTS and SD

To investigate the association between neurological impairment and LUTS, patients were stratified according to their neurological disability based on the EDSS score. Patients were also compared in terms of the total ICSmSF score and the incontinence/storage subscore (ICSI). The EDSS scores were correlated with the IIEF-15 scores to evaluate the influence of the degree of neurological impairment on sexual function. Finally, we investigated the direct association between LUTS and SD by correlating ICSmSF and IIEF-15 scores.

### Statistical Analysis and Ethical Considerations

Continuous or ordinal quantitative variables are described by measures of central tendency (mean or median) and the respective measures of dispersion (standard deviation or interquartile range). Qualitative or categorical variables are described using their absolute values or proportions. Student's t test, the Mann-Whitney test, the chi-squared test or Fisher's test were used as appropriate. The Pearson correlation test was used to evaluate correlations between continuous variables. Statistical significance was defined as *p*<0.05. Data were processed using commercially available statistical software (Graph Pad Prism version 5.0.3, Graph Pad Software, San Diego, CA). This study was approved by the Institutional Review Board of our hospital.

## RESULTS

### Study Population

A total of 41 men with an average age of 40.7±10.1 years (ranging from 22 to 66 years) were evaluated. Thirty-five (85.4%) patients were Caucasian, and 6 (14.6%) were African-Brazilians. The mean disease duration was 10.5±7.3 (5-35) years. The median EDSS score was 3.0 [range of 1.5 to 6]. Among the patients, 25 (61%) had scores between 1.0 and 4.5, and 16 (39%) had scores ≥5.0. Demographic characteristics are shown in Table 1.

### Prevalence of LUTS

The overall prevalence of LUTS was 97.6%, with only one patient presenting no symptoms. The most common storage symptoms were urgency (70.7%), increased daytime frequency (46.3%), urge urinary incontinence (36.6%), nocturia (31.7%), unpredictable urinary incontinence (21.9%), nocturnal enuresis (19.5%), and stress urinary incontinence (9.8%). Hesitancy was the most common voiding symptom (70.7%), followed by intermittency (46.3%), feeling of incomplete emptying (43.4%), weak stream (41.4%) and straining to void (36.6%).

**Figure 1:**
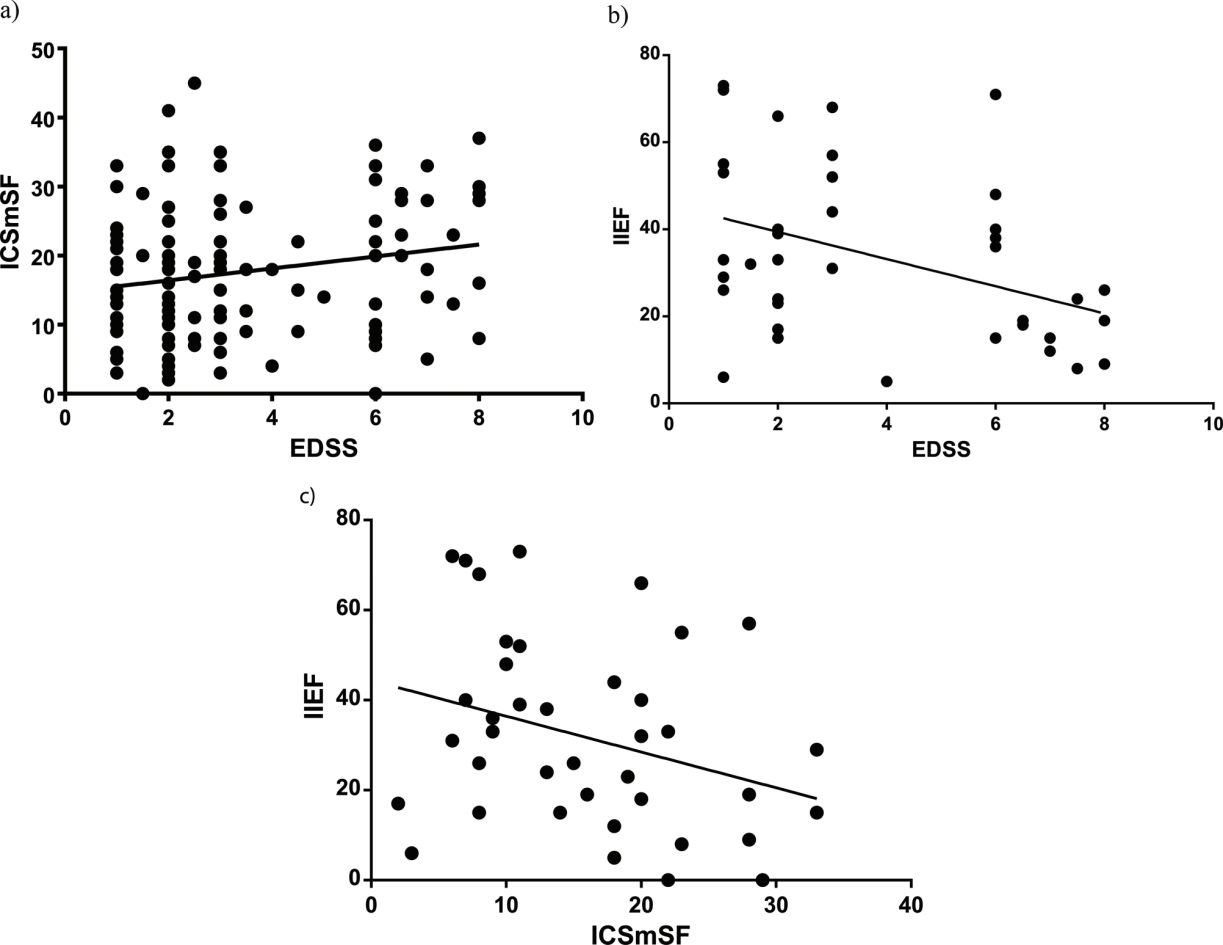
**(a)** Association between neurological impairment (EDSS) and lower urinary tract symptoms (ICSmSF) (r=0.16 [0.02 to 0.36] *p*=0.29). **(b)** Association between neurological impairment (EDSS) and sexual function (IIEF-15) (r=-0.41 [-0.65 to -0.11]; *p*=0.008). **(c)** Association between lower urinary tract symptoms (ICSmSF) and sexual function (IIEF-15) (r=-0.31 [-0.56 to -0.01]; *p*=0.04).

Regarding the impact of LUTS on QOL, 11 (26.8%) patients reported no bother, 22 (53.7%) reported mild to moderate burden, and 8 (19.5%) reported severe burden. The prevalence of LUTS and the impact on QOL are shown in Table 2. A significant association between the severity of LUTS and the impact of LUTS on QOL was found. The highest correlation coefficient was observed with symptoms of incontinence (Figure 1). Table 3 compares the prevalence of LUTS in our study of patients with MS compared to the general population reported in the EpiLUTS study 15.

Urinary tract sonography was normal in 37 (90.2%) patients, while four (9.8%) patients had kidney cysts. Prostate transabdominal sonography demonstrated a PVR >100 ml in 7 (17.1%) patients. The mean serum creatinine level was 0.77± 0.17 mg/dl (range 0.63–1.2), and the mean serum PSA level was 0.64±0.11 ng/ml (range 0.1-1.98). Urine culture was positive in 8 (19.5%) patients.

### Prevalence of SD

A total of 39 (95.1%) men reported being sexually active. Their median IIEF-15 score was 29 [15-46]. A total of 29 (74.4%) had SD (IIEF-15≤45). Significant ED (IIEF-5≤16) was reported by 26 (66.7%) patients, including severe ED in 17 (43.6%) and moderate ED in 9 patients (23.1%). Orgasmic dysfunction was graded as severe in 14 (35.9%) patients, moderate in 5 (12.8%) patients, mild to moderate in 5 (12.8%) patients and mild in 7 (18%) patients; 8 (20.5%) patients reported normal orgasmic function. The decrease in sexual desire was classified as severe in 2 (5.1%) patients, moderate in 11 (28.2%) patients, mild to moderate in 4 (10.3%) patients and mild in 10 (25.6%) patients; 12 (30.8%) patients reported normal sexual desire. Twenty-three (59%) patients reported moderate or severe dissatisfaction with sexual intercourse (23.1% and 35.9%, respectively), and 3 (7.7%) considered themselves satisfied. Regarding the overall satisfaction with sexual life, 11 (28.2%) patients considered themselves as severely dissatisfied, 10 (25.6%) considered themselves moderately dissatisfied, 9 (23.1%) considered themselves mildly to moderately dissatisfied, and 3 (7.7%) considered themselves mildly dissatisfied; 6 (15.4%) patients were satisfied. Table 4 summarizes the prevalence of SD according to IIEF domains.

### Associations among Neurological Impairment and LUTS and SD

Patients with more severe neurological impairment (EDSS ≥4.5) had worse storage symptoms according to the ICSI score (6 [4-10] *vs*. 4 [2-6]; *p*=0.014), while the total ICSmSF score did not differ significantly (17 [11-23] *vs* 13 [8-21]; *p*=0.267). A significant correlation was found between the EDSS score and the IIEF-15 score (r=-0.41 [-0.65 to -0.11]; *p*=0.008), demonstrating that higher degrees of neurological deficit are associated with a higher prevalence of SD. The association between the EDSS and IIEF-15 scores is displayed in Figure 1. A correlation was observed between the severity of LUTS and the presence of SD (r=-0.31 [-0.56 to -0.01]; *p*=0.04), demonstrating that more severe LUTS are associated with worse sexual function.

## DISCUSSION

In this cross-sectional study, we evaluated a population of men with MS to determine the prevalence and characteristics of LUTS and SD and their relationship to neurological impairment. LUTS were present in all but one man in our patient population (97.6%), based on the definition used in most population-based studies: symptoms occurring sometimes, most of the time or all of the time. Storage symptoms were reported by 85% of the patients and represented the most common ICS category of symptoms, with urgency (70.7%), frequency (46.3%) and urge urinary incontinence (36.6%) as the most common symptoms. Voiding symptoms were present in 72.5% of the patients, with hesitancy (70.7%), intermittency (46.3%) and feeling of incomplete emptying (43.4%) as the most prevalent symptoms. Symptoms were commonly bothersome: 53.7% of patients were mildly to moderately burdened and 19.5% of patients were severely burdened by LUTS. As expected, symptom severity was associated with symptom burden.

Studies have shown that most patients with MS will develop LUTS during the course of the disease 20,21. According to a database of the North American Research Committee on Multiple Sclerosis with more than 9700 patients, 65% report moderate to severe LUTS 22. Storage symptoms are the most frequent in patients with MS 23. Urgency, frequency and urge incontinence can be present in 19 to 85% of patients 24,25. The reported prevalence of voiding symptoms in MS patients is 37-72% 5,26-29. The overall prevalence of LUTS in our sample of men with MS (97.6%) was comparable to that in other series (52-97%) 5,26, and storage symptoms were the most common LUTS. We also found a high prevalence of voiding symptoms, which have been shown to be more common in men 5,29. Considering differences in study populations and methods of evaluating LUTS, our findings appear consistent with other studies. Our observation that LUTS affects the QOL of most MS patients is consistent with other studies that have shown that LUTS are among the most uncomfortable and limiting symptoms that affect these patients, as they deteriorate their work and social activities, self-esteem and psychological status 12,26.

Most subjects (95.1%) in our study were sexually active. Their sexual function, however, was markedly affected in many domains, with 3 of every four patients classified as having SD (IIEF-15≤45). ED (IIEF-5≤16) was reported by two-thirds of the patients, with severe ED in 43.6%. Moderate or severe orgasmic dysfunction was present in 48.7% of the patients, with only 20.5% reporting normal orgasmic function. Decreased sexual desire was also common and was moderate or severe in 33.3% of patients, while only 30.8% reported normal sexual desire. Most patients (59%) reported moderate or severe dissatisfaction with sexual intercourse, and only 7.7% considered themselves satisfied. In terms of satisfaction with sexual life, 53.8% of subjects considered themselves as moderately or severely dissatisfied, and only 15.4% were satisfied.

As with LUTS, SD also has a tremendous negative impact on the QOL of people with MS 8. Our findings for SD are consistent with most studies, which have demonstrated that 50-90% of men with MS have SD 26. ED is reported as the most common problem, followed by ejaculatory and/or orgasmic dysfunction and reduced libido 9. We observed significant impairment of EF, orgasm and sexual desire. Accordingly, patients reported low rates of satisfaction with sexual life.

LUTS and SD have been shown to be correlated in individuals with or without neurological diseases 11,18, reinforcing the concept of a common etiology for both dysfunctions. MS results in a demyelination process that interrupts the continuity of the neural pathways, which has the potential to substantially alter the neurophysiology of both the lower urinary tract function and sexual function. Our findings corroborate the association between LUTS and SD in MS, based on the significant correlation between the ICSmSF and the IIEF-15 scores. Voiding dysfunction has been shown to be independently associated with SD after adjustment for psychological factors in a multivariable model 30. Francomano et al. 31 investigated the impact of daily tadalafil treatment on LUTS and SD in men with MS and demonstrated significant improvements in storage symptoms, PVR volume and EF. In an Italian study with 101 male patients, Balsamo et al. demonstrated that depression and urinary symptoms measured by the International Prostate Symptom Score (IPSS) were the only independent predictors of ED in this study population 32. Finally, urodynamic abnormalities such as detrusor overactivity and low bladder capacity have been shown to correlate with SD in both men and women.

The severity of LUTS has been shown to correlate with the degree of neurological impairment in MS patients 5,12 and other neurological diseases such as Parkinson's Disease 33 and neuromyelitis optica 34. Zecca et al. evaluated 403 men and women with MS and found that urinary incontinence has a strong positive correlation with EDSS scores in both genders 4. Araki et al. showed that storage but not voiding symptoms correlated with EDSS scores 35. Our findings were consistent with these results, confirming the association between neurological impairment and storage symptoms measured by the ICSI score.

SD appears to be highly correlated with the severity of neurological impairment, affecting approximately 80% of patients with an EDSS score >4.0 10. Our results confirm this association, showing a significant correlation between the EDSS and the IIEF-15 scores. The pathophysiology of SD in patients with MS is multifactorial, involving physical, psychological and social factors 36. Marck et al. 36 demonstrated that SD and low satisfaction with sexual life are associated with depression and fatigue, as well as with modifiable lifestyle factors such as diet and physical activity. Fragala et al. 18 found that, in addition to neurological disability, depression and the presence of detrusor overactivity were independent predictors of SD in men and women.

The strengths of this study include the evaluation of both LUTS and SD in men using well-established, validated diagnostic tools with definitions of symptoms based on the ICS definitions of LUTS. Moreover, questionnaires were completed in the setting of a medical evaluation and not based on only self-reported responses or on telephone interviews during which subjects may not always provide accurate answers. The relatively small sample size is a limitation of the study, but it must be noted that we included only men. Another limitation of the study was that we did not control for the possible effects of medications used for MS treatment on LUTS or SD. Because patients were using several medications from different drug classes, our sample would be underpowered to analyze their effects.

## CONCLUSION

LUTS and SD are highly prevalent in men with MS, and both have a negative impact on QOL. The severity of neurological impairment correlates with the severity of LUTS and SD. Given the high prevalence of LUTS and SD in men with MS, a more attentive focus on these areas is essential to help patients achieve better control of LUTS and improve their sexual health with consequent benefits to their QOL.

## AUTHOR CONTRIBUTIONS

We confirm that all authors have made substantial contributions to the conception, design, drafting and critical revision of the manuscript for important intellectual content and provided final approval of the version to be published.

## Figures and Tables

**Table 1:** Demographic and clinical data of male patients with MS.

Age	40.7±10.1 (range 22-66) years
Race	
Caucasian	35 (85.4%)
African-Brazilian	6 (14.6%)
Other	0
Marital status	
Married/living with partner	21 (48.8%)
Single	14 (34.2%)
Divorced	6 (14.6%)
Widower	0
MS duration	10.5±7.3 (range 5-35) years
EDSS median	3.0 (1.5-6)

**Table 2:** LUTS in men with MS.

LUTS - Storage symptoms	*n* (%)
Urgency	29 (70.7%)
Increased daytime frequency	19 (46.3%)
Urge urinary incontinence	15 (36.6%)
Nocturia	13 (31.7%)
Continuous urinary incontinence	9 (21.9%)
Nocturnal enuresis	8 (19.5%)
Stress urinary incontinence	4 (9.8%)
LUTS - Voiding symptoms	
Hesitancy	29 (70.7%)
Intermittent stream	19 (46.3%)
Slow stream	17 (41.4%)
Straining	15 (36.6%)
LUTS - Postmicturition	
Incomplete emptying	18 (43.4)
ICSmSF median	15 (9-22)
ICSI	5.6±0.8
ICSV	6.8±0.4
ICSF	1.4±0.7
Nocturia	1.0±0.7
Quality of life (QOL)	
Not bothered	11 (26.8%)
Mildly bothered	18 (43.9%)
Moderately bothered	4 (9.8%)
Severely bothered	8 (19.5%)

**Table 3:** Prevalence of LUTS in men with MS found in the present study in comparison to the general population reported in the EpiLUTS study.

Condition, *n* (%)	Men with MS (present series)		Men in the general population (EpiLUTS study)	
	*LUTS at least “sometimes”*	*LUTS at least “most often”*	*LUTS at least “sometimes”*	*LUTS at least “often”*
No. of participants	41	41	14,139	14,139
**Voiding symptoms**				
Weak stream	15 (36.6)	9 (21.9)	3,803 (27.0)	1,356 (9.6)
Split stream	-	-	2,743 (19.5)	694 (4.9)
Intermittency	19 (46.3)	9 (21.9)	2,632 (18.7)	721 (5.1)
Hesitancy	29 (70.7)	9 (21.9)	2,833 (20.1)	751 (5.3)
Straining	15 (36.6)	5 (12.2)	1,092 (7.7)	275 (2.0)
Terminal dribble	-	-	6,410 (45.5)	2,755 (19.6)
**Storage symptoms**				
Perceived frequency, yes	19 (46.3)	-	2,895 (20.5)	2,895 (20.5)
Nocturia ≥1	27 (65.8)	-	9,788 (69.4)	-
Nocturia ≥2	13 (31.7)	-	4,021 (28.5)	-
Nocturia ≥3	-	-	-	1,825 (12.9)
Urgency	29 (70.7)	12 (29.3)	3,104 (22.4)	674 (4.9)
Urgency with fear of leaking	-	-	1,975 (14.0)	546 (3.9)
Urgency incontinence	15 (36.6)	1 (2.4)	1,315 (9.3)	639 (4.5)
**Stress incontinence**				
Laughing, sneezing, coughing	4 (9.7)	2 (4.9)	163 (1.2)	63 (0.4)
Physical activities	-	-	183 (1.3)	85 (0.6)
Leak for no reason	9 (21.9)	2 (4.9)	196 (1.4)	124 (0.9)
Nocturnal enuresis	8 (19.5)	3 (7.3)	201 (1.4)	116 (0.8)
Leak during sexual activity	-	-	36 (0.3)	11 (0.1)
**Postmicturition**				
Incomplete emptying	18 (43.9)	5 (12.2)	3,209 (22.7)	764 (5.4)
Postmicturition incontinence	18 (43.9)	3 (7.3)	4,202 (29.7)	2,333 (16.5)

**Table 4:** Sexual Dysfunction in men with MS.

**Sexual Function**	n=39
SD	29 (74.4%)
No SD	10 (25.6%)
**IIEF-15 median**	29 (15-46)
Erectile dysfunction	26 (66.7%)
Orgasmic dysfunction	19 (48.7%)
Decreased sexual desire	13 (33.3%)
Dissatisfaction with sexual intercourse	23 (59%)
Overall satisfaction with sexual life	21 (53.8%)

MS = multiple sclerosis; EDSS = Expanded Disability Status Scale; LUTS = lower urinary tract symptoms; ICSmSF = International Continence Society male short form; ICSI = International Continence Society male short form storage symptoms (incontinence); ICSV = International Continence Society male short form voiding symptoms; ICSF = International Continence Society male short form frequency; QOL = quality of life; SD = sexual dysfunction; IIEF-15 = International Index of Erectile Function-15.
